# Machine Learning-Based Composition Design of Functionally Graded Alloys

**DOI:** 10.3390/ma19102174

**Published:** 2026-05-21

**Authors:** Yimao Yu, Yiqing Wang, Pu Zhao, Boyu Zhang, Yuan Huang

**Affiliations:** 1 School of Materials Science and Engineering, Tianjin University, Tianjin 300350, China; 15574834986@163.com (Y.Y.); wangyiqing@tju.edu.cn (Y.W.); 18503222579@163.com (B.Z.); 2 Shagang School of Iron and Steel, Soochow University, Suzhou 215021, China; 20245249007@stu.suda.edu.cn

**Keywords:** composition design, functionally graded materials, machine learning, SHAP analysis

## Abstract

Functionally graded materials (FGMs) effectively alleviate residual stress induced by physical property mismatch at dissimilar material interfaces through a graded transition in composition or structure. Among these, the matching of the coefficient of thermal expansion (CTE) is a core indicator for ensuring the service reliability of the joint. Traditional composition design relies on empirical trial-and-error, which makes it difficult to efficiently identify the optimal path in a high-dimensional composition space. This study proposes a data-driven, machine learning-assisted composition design method. Based on a high-precision dataset covering 15 elements and 747 CTE data points, six typical regression models were systematically evaluated. The results show that the random forest (RF) model achieves the best performance, with a coefficient of determination (*R*^2^) of 0.929 and a root mean square error (*RMSE*) of 0.658 on the test set. Using the SHapley Additive exPlanations (SHAP) method, the lattice constant (*c*), Young’s modulus (*YM*), and temperature (*T*) were identified as the key physical descriptors governing the thermal expansion behavior. Experimental validation shows that the CTE prediction deviation of the model for the high-performance Fe-based alloy Norem02 in the range of 20–300 °C is only 0.89%. Based on this framework, the composition of the 316L/Norem02 transition layer was successfully designed in this study. This effectively reduced the interfacial thermal expansion mismatch. Consequently, it provides a reliable theoretical basis for the rational design of dissimilar material interfaces under extreme service conditions.

## 1. Introduction

In industries such as power generation, aerospace, high-pressure energy equipment, and power systems, components are often required to possess high strength, excellent wear and corrosion resistance, and superior thermophysical stability. Since a single monolithic material can hardly satisfy all these requirements simultaneously, the joining and compounding of dissimilar materials have become a common choice in industry [1,2]. However, when two materials are joined or laminated, factors such as a mismatch in the coefficient of thermal expansion (CTE) often lead to failure behaviors in the resulting joint or laminated composite, including cracking and delamination. For example, Naffakh et al. [3] investigated the dissimilar welding of AISI 310 austenitic stainless steel and Inconel 657 nickel-based superalloy. They found that the thermophysical property mismatch between the base metals couples with the solidification cracking susceptibility of the weld metal. This significantly reduces the service reliability of the component. To address this issue, researchers typically introduce a transition layer at the interface to form functionally graded materials (FGMs), thereby achieving stress regulation. Sun et al. [4] introduced a W foil as a low-expansion transition layer in the joining of Invar alloy and SiO_2_f/SiO_2_ composite. This reduced the CTE mismatch. The resulting joint achieved a shear strength of 33 MPa, which is 1.75 times that obtained using a single AgCuTi brazing filler metal.

FGMs are a class of advanced materials whose structure or composition varies continuously with spatial position according to a specific pattern to meet the requirements of extreme service environments. They hold significant application prospects in fields such as nuclear energy, aerospace, and marine engineering [5,6,7]. The introduction of a gradient structure can significantly reduce the residual stress level in the joint region, thereby improving the mechanical properties and physical functional stability. It is worth noting that the magnitude of residual stress is not only related to the interlayer interface properties but is also directly influenced by the distribution of intralayer characteristics [8]. Therefore, how to efficiently and accurately design the composition of the gradient transition layer to meet the target CTE profile is one of the core issues in FGM application research.

Traditional FGMs composition design relies mainly on experimental trial-and-error and experience-oriented qualitative judgments. Researchers often need to repeatedly prepare and test a large number of transition layers with different compositions. This process is time-consuming and costly, making it difficult to meet the urgent demands for efficiency and accuracy in modern materials research and development. In recent years, advances in machine learning (ML) have revolutionized research in materials science. Its effectiveness in optimizing material compositions and predicting properties has been extensively validated. By deeply mining the high-dimensional data generated by the Materials Genome Engineering, this technology efficiently elucidates the nonlinear relationships between composition, processing, and properties in multi-component systems. It thus offers a data-driven, innovative strategy for the targeted design of new high-performance materials. Research indicates that machine learning-based high-throughput screening methods can overcome the efficiency bottleneck of traditional trial-and-error approaches. They significantly accelerate the materials development process and open up new avenues for revealing the structure-property relationships of complex material systems [9,10,11]. Tang et al. [12] successfully discovered a Ni-based superalloy with low thermal expansion by using a support vector regression (SVR) model to predict its thermal expansion. Liu et al. [13] proposed a machine learning-enhanced Scheil–Gulliver model method. By developing phase classification and composition prediction models combined with parallel algorithms, they achieved high-throughput solidification simulations. They also introduced a data-driven linear gradient path planning algorithm, providing an efficient tool for FGMs design. Chen et al. [14] proposed a probabilistic physics-guided neural network to analyze the probabilistic fatigue behavior of L-PBF Ti6Al4V by considering the influence of processing parameters. Liu et al. [15] constructed a model using image recognition technology and the XGBoost algorithm, successfully predicting the Young’s modulus and elongation of materials. The above studies have validated the great potential of ML in the prediction and design of alloy properties.

In the composition design of transition layers for dissimilar material joints, the CTE is a key control indicator. To this end, this study constructed a multi-component alloy dataset covering 15 key elements and containing 747 CTE data points. On this basis, through systematic feature engineering and algorithm optimization, a machine learning model capable of accurately predicting the relationship between alloy composition and CTE was developed. The SHAP method was then employed to reveal the key physical factors influencing CTE. Finally, taking the dissimilar joining of 316L stainless steel and Norem02 alloy as an example, the established model was used to guide the composition design of the intermediate transition layer. The engineering feasibility of this method was then verified through experiments. This work not only confirms the practical value of machine learning in accelerating the composition design of functionally graded materials, but also provides a referable methodology for the rational design of other dissimilar material interfaces.

## 2. Materials and Methods

### 2.1. Machine Learning Model Framework

**Figure 1:**
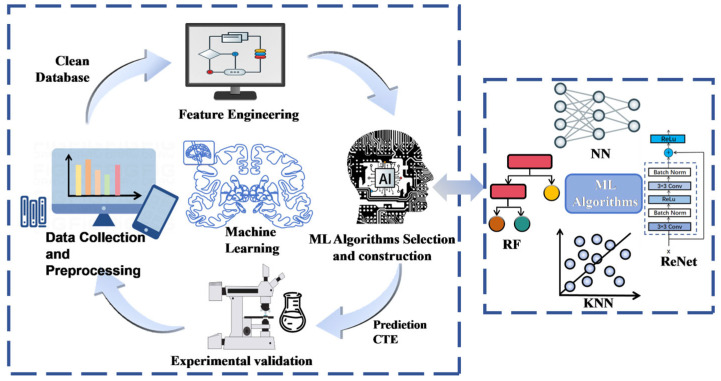
Flowchart for constructing the relationship between alloy composition and CTE using ML.

The framework for designing an ML model to establish the composition–CTE relationship for common metallic materials (involving elements such as C, Cr, Ni, Mn, Mo, Fe, Al, Co, Cu, W, Nb, Ta, V, Ti and Si) consists of four main components: data collection, feature engineering, selection of ML algorithms and construction of the composition–CTE relationship. This process is illustrated in Figure 1.

#### 2.1.1. Data Collection

The collected database was sourced from published literature, databases, and MatWeb. The following preprocessing steps were applied to the raw data: data points with obvious entry errors or low source credibility were removed; for samples with identical composition and duplicate CTE values, the arithmetic mean was taken as the final representative value for that composition; incomplete samples missing key fields were deleted. After the above screening, a dataset containing 747 valid CTE data points was finally constructed. The dataset covers 15 common alloying elements (C, Si, Cr, Ni, Mn, Mo, Fe, Al, Co, Cu, W, Nb, Ta, V, Ti). Fe-based alloys account for approximately 90% of the data, while Ni-based and Co-based alloys together account for about 10%. Regarding the temperature distribution, there are 200 data points at or below 300 °C, 537 data points in the range of 300–800 °C, and 10 high-temperature data points above 800 °C. The model training adopted a random split of 80% training set and 20% test set. In this work, the focus is on the effect of alloy composition on CTE rather than on specific alloy manufacturing processes. This choice is justified because CTE is primarily governed by composition and is influenced to a lesser extent by process variations in alloy manufacturing [16]. To eliminate the adverse effects of dimensional differences among different feature parameters on model training, the raw data were preprocessed using Z-score normalization before feature engineering. This method transforms the values of each feature into a standard normal distribution with a mean of 0 and a standard deviation of 1. The transformation formula adopts Equation (1).

(1) $$x^{\prime }=\frac{x_{i}-\bar{x}}{\sigma_{x}}$$

In this equation, $x_{i}$ is the value of the original feature x for the i-th sample, while $\bar{x}$ and *σ_x_* are the mean and standard deviation of feature x, respectively. After this transformation, all features are placed on a comparable scale. This effectively prevents features with larger numerical magnitudes from dominating the weights during model training, thereby improving the convergence stability of gradient descent and the prediction accuracy of the model.

To comprehensively assess the boundaries of the model’s predictive capability, careful consideration must be given to its applicability to special alloy systems. Since the training dataset in this study was primarily constructed based on the thermal expansion behavior of conventional alloys (Fe-based, Ni-based, and Co-based alloy systems), the current model framework does not possess predictive reliability for systems with fundamentally different physical mechanisms, such as Invar alloys. The training samples of this model cover a limited temperature range (only 10 data points above 800 °C, with the remaining data all below 800 °C). The CTE prediction accuracy is guaranteed only within the learned temperature interval. When extrapolating to temperatures far above or far below the range covered by the training data, particularly near the alloy phase transformation point or the service limit temperature, the prediction deviation may increase significantly. Finally, the model primarily analyzes the dominant effect of chemical composition on CTE and does not incorporate processing parameters. Therefore, for scenarios where process conditions significantly alter the thermal expansion behavior, the prediction results may contain certain errors.

#### 2.1.2. Feature Engineering

In machine learning modeling, the selection of feature descriptors is crucial to model performance. As the fundamental units in a dataset, these feature values directly reflect the intrinsic properties and underlying patterns of the data. Data and features determine the upper bound of ML performance, whereas models and algorithms can only approach this bound. In this work, the candidate descriptors encompass a range of atomic parameters that have demonstrated excellent performance across various problems [17,18,19], as well as phase parameters, including valence electron concentration (*VEC*) [20], atomic size difference (*δ*) [21], enthalpy of mixing (Δ*H_mix_*) [21], entropy of mixing (Δ*S_mix_*) [22], average melting point (*T_m_*) and solid solution phase formation parameter (*Ω*) [23]. To calculate the above parameters, this study employed a multi-component thermodynamic calculation software independently developed by our research group (Software Copyright Registration No. 2021SR1404650). The core algorithm of this software is based on an improved Miedema model and an extrapolated extension of the Chou kinetic model [24]. During the research, all commonly used feature values were compiled and organized. The details are provided in Tables S1 and S2 of the Supplementary Information. A total of 42 initial descriptors were obtained.

Feature selection is the next and critical step in the machine learning process after preparing the raw feature pool. The purpose of feature selection is to obtain the most representative subset from the raw feature pool, which should contain essential information with less redundancy. By employing feature selection, the computational time and the risk of overfitting can be reduced, while the interpretability of the model can be enhanced to some extent [25].

**Figure 2:**
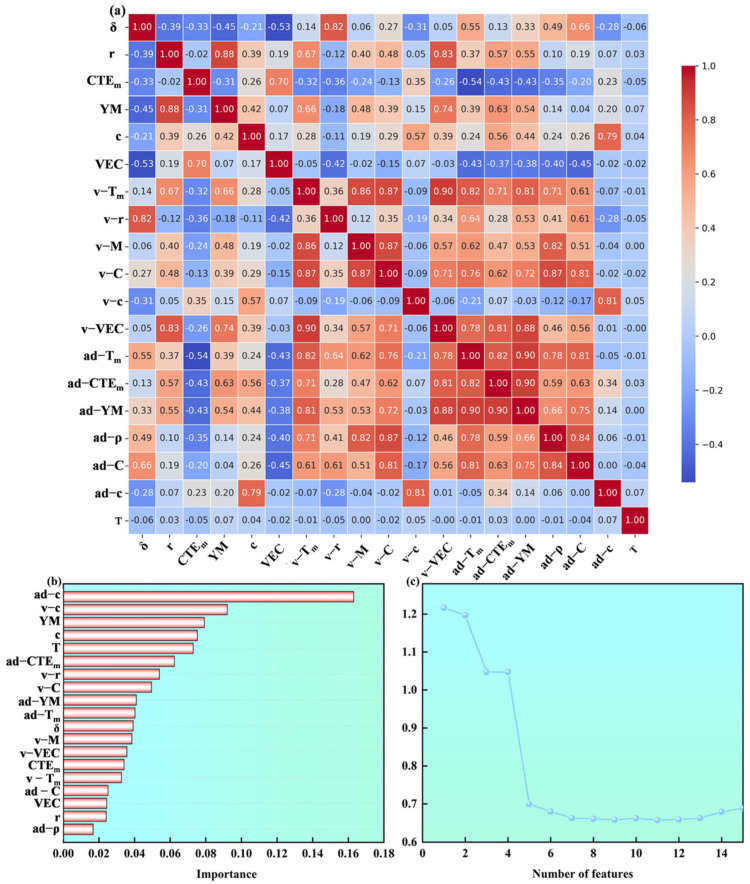
(**a**) Pearson correlation coefficient; (**b**) Feature importance scores based on decision tree splitting frequency in RF algorithm; (**c**) Recursive Feature Elimination.

This study adopted a three-step feature selection method [26] (Figure 2). First, the Pearson correlation coefficient (PCC) between features was calculated. Features with an absolute PCC value greater than 0.9 were removed to eliminate strong correlations [27,28,29]. The results are shown in Figure 2a. Second, the RF algorithm was used to evaluate the importance scores of the remaining features (Figure 2b). An importance threshold was set, and features with scores below this threshold were excluded, retaining those with significant predictive contributions to the CTE. Finally, based on the feature subset obtained in the second step, a recursive feature elimination (RFE) strategy was employed. At each iteration, the feature with the lowest importance was removed until the model performance reached its optimum (Figure 2c). A key feature subset that is most representative for predicting the coefficient of thermal expansion was ultimately screened out.

#### 2.1.3. Selection of ML Algorithms

Six machine learning algorithms [30] were employed for feature selection, and the best-performing algorithm was concurrently identified. These algorithms include random forest (RF) [31], linear Regression (LR) [32], multi-layer perceptron (MLP) [33], K-nearest neighbors (KNN) [34], neural network (NN) [35,36,37], and residual neural network (ReNet) [38]. Scikit-learn [39] is a leading machine learning library that integrates numerous cutting-edge and widely adopted machine learning algorithms, providing a powerful toolkit for data scientists and researchers to address medium-scale supervised and unsupervised learning challenges. All algorithms used in this work were implemented within the scikit-learn framework. During the ML algorithm selection stage, the preprocessed dataset was split into a training set (80%) and a test set (20%). Throughout the model training process, multiple hyperparameters were systematically fine-tuned, and 10-fold cross-validation was employed to precisely evaluate model performance. All hyperparameter settings are included in Part II of the Supplementary Information. ML model construction is a process of hyperparameter search, primarily conducted using cross-validation (CV) methods. The 10-fold cross-validation [40] (10-fold CV) method minimizes the impact of overfitting in nonlinear regression, thereby making full use of the data and enhancing the predictive capability of the model [41]. Finally, different machine learning algorithms were used to train on these features. The final feature set and the optimal ML algorithm were determined based on the *R*^2^ and the *RMSE*. *R*^2^ and *RMSE* are calculated by Equations (2) and (3), respectively.

(2) $$R^{2}=1-\frac{\sum_{i=1}^{n}{\left(y_{i}-{\hat{y}}_{i}\right)}^{2}}{\sum_{i=1}^{n}{\left(y_{i}-\bar{y}\right)}^{2}}$$

(3) $$RMSE=\sqrt{\frac{\sum_{i=1}^{n}{\left(y_{i}-{\hat{y}}_{i}\right)}^{2}}{n}}$$

where *n* is the number of data points in the test set, *y* is the true value, *ŷ* is the predicted value, and $\bar{y}$ represents the mean value.

### 2.2. Experimental Materials

In the experiment, the chemical compositions of the Norem02 alloy and 316L stainless steel used are listed in Table 1. The laser was a YLS-2000-TR fiber laser manufactured by IPG Photonics (Burbach, Germany). The powder feeder was a GTV model PF2/2M. The main process parameters for fabricating the graded composite material were as follows: laser power of 1000 W, powder feed rate of 0.6 r/min, carrier gas flow rate of 2.0 L/min, and scanning speed of 6 mm/s. Both the carrier gas for the powder and the shielding gas were argon. The CTE was measured using a thermomechanical analyzer (Netzsch DIL 302PC, Netzsch Gerätebau GmbH, Selb, Germany). The sample dimensions were Φ18 mm × 6 mm. According to the ASTM E228 standard [42], the sample was heated to 500 °C at a heating rate of 5 °C/min.

## 3. Results and Discussion

### 3.1. Selection of Machine Learning Algorithms

To establish the optimal model architecture for predicting the CTE of alloys, this study systematically compared the regression performance of six typical machine learning algorithms on the same dataset, including RF, linear regression (LR), multi-layer perceptron (MLP), K-nearest neighbors (KNN), neural network (NN), and residual neural network (ReNet). Each model adopted the optimal feature subset obtained after feature selection as input variables (the complete feature subsets and hyperparameter configurations corresponding to the models are detailed in Tables S3 and S4 of the Supplementary Information), and hyperparameter optimization was performed using 10-fold cross-validation and grid search strategies to ensure the reliability and comparability of the evaluation results.

**Figure 3:**
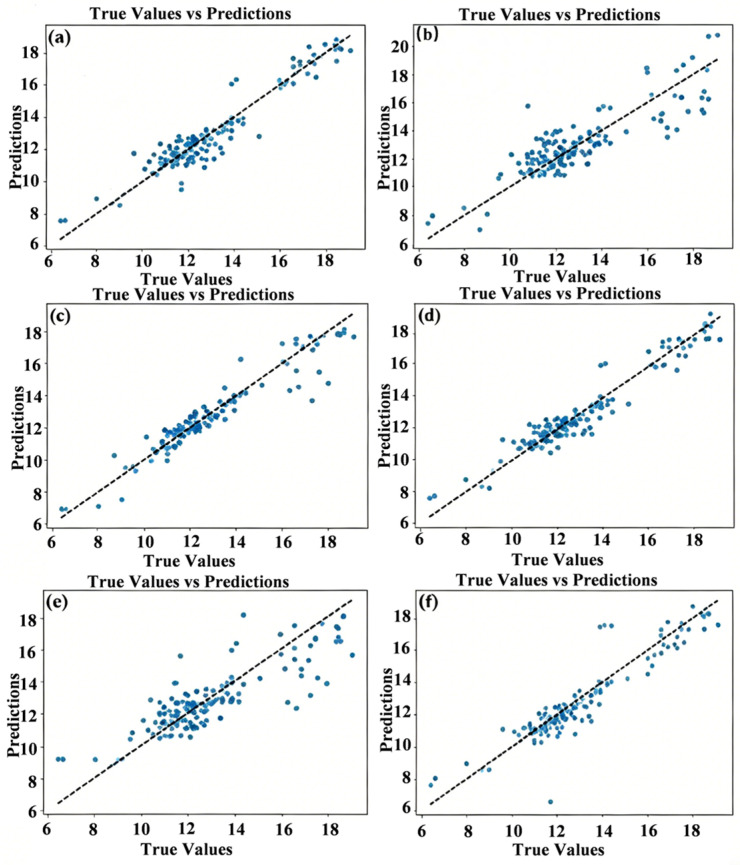
Prediction performance of prediction models: (**a**) KNN, (**b**) LR, (**c**) NN, (**d**) RF, (**e**) MLP, (**f**) ReNet.

Figure 3 presents the scatter plots of predicted versus true values on the test set for the six algorithms, where the horizontal axis represents the experimental CTE values and the vertical axis represents the model-predicted values. The diagonal line (*y* = *x*) represents ideal unbiased predictions. Among them, the scatter points of the RF model are tightly distributed around the diagonal line, exhibiting the smallest overall deviation.

**Figure 4:**
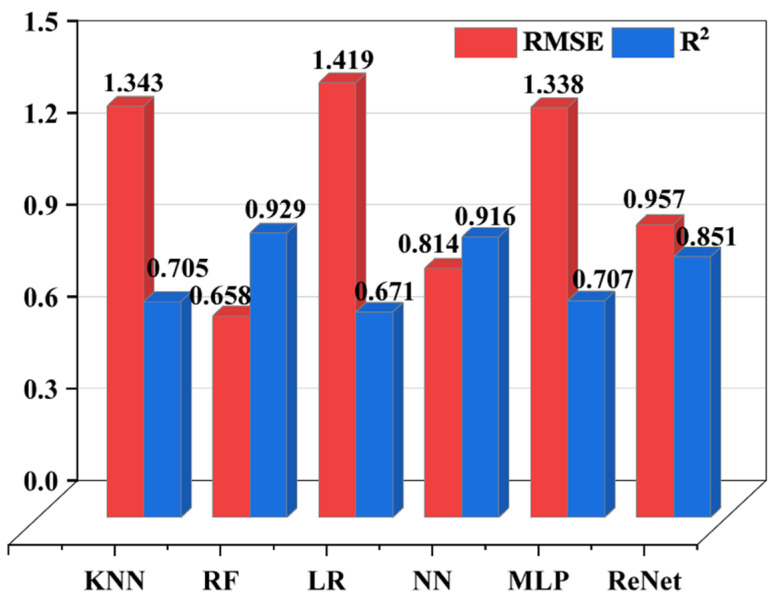
Fitting results of different algorithms to the data.

Furthermore, Figure 4 shows a comparison of the prediction accuracy of each algorithm on the test set. Considering the two key evaluation metrics, *R*^2^ and *RMSE*, the RF algorithm exhibits the best overall performance in the CTE prediction task. The key features for the RF algorithm are *ad-T_m_*, *δ*, *YM*, *c*, *v*-*r*, *v*-*C*, *v*-*c*, *ad*-*CTE_m_*, *ad*-*YM*, *ad*-*c* and *T*. It achieves an *R*^2^ of 0.929 and an *RMSE* of only 0.658. In contrast, the other algorithms are inferior to RF in terms of both prediction accuracy and generalization capability. This result can be attributed to the good adaptability of the ensemble learning mechanism of RF to the nonlinear coupling relationships between alloy composition and properties.

Based on the above analysis, this study selected the RF model as the fundamental algorithmic framework for subsequent CTE prediction and key factor interpretation.

### 3.2. Experimental Validation of Model Accuracy

**Figure 5:**
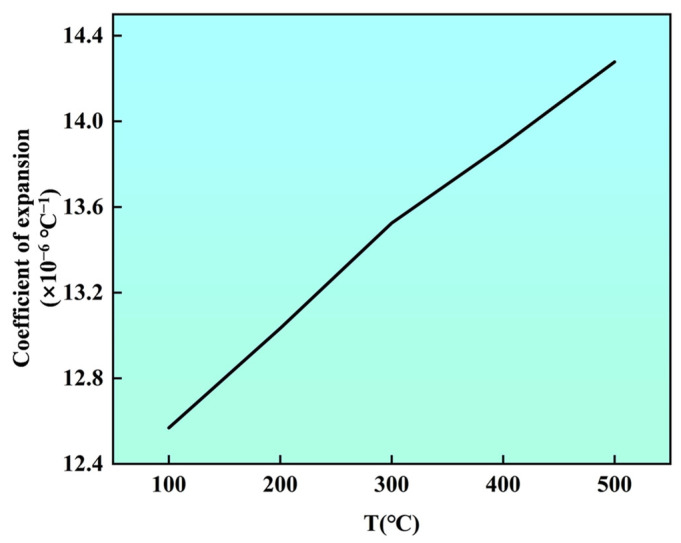
Experimentally determined CTE of Norem02 alloy.

To validate the generalization ability and prediction reliability of the established RF model in a real alloy system, this study selected Norem02 alloy as the experimental validation material. The experimental measurement results of the CTE for this alloy are shown in Figure 5. For quantitative comparison, the CTE value within the characteristic temperature range of 20–300 °C was selected as the evaluation basis. As shown in Table 2, the model-predicted CTE value of the Norem02 alloy is 13.4034 × 10^−6^ °C^−1^, while the experimentally measured value is 13.5251 × 10^−6^ °C^−1^. The relative deviation between the two is only 0.89%. Considering the inherent dispersion of the CTE due to micro-compositional fluctuations and testing conditions, this deviation is well within an acceptable range.

The above results fully demonstrate that the established data-driven RF regression model can accurately capture the complex nonlinear relationship between alloy composition and the CTE. It exhibits excellent prediction accuracy for the thermal expansion behavior of multi-component alloy systems. This validation further confirms that the model can provide a reliable theoretical basis for the computational design of alloy compositions.

### 3.3. Relationship Between Key Factors and CTE

To thoroughly elucidate the relationship between the features and the CTE, the SHAP method was introduced to perform a global interpretability analysis of the model [43]. This method is based on the concept of Shapley values from cooperative game theory. By calculating the marginal contribution of each feature to the prediction outcome, it can effectively quantify the importance and direction of influence of each input variable in the “black-box” model, thereby revealing the key physical factors affecting the thermal expansion behavior of alloys.

**Figure 6:**
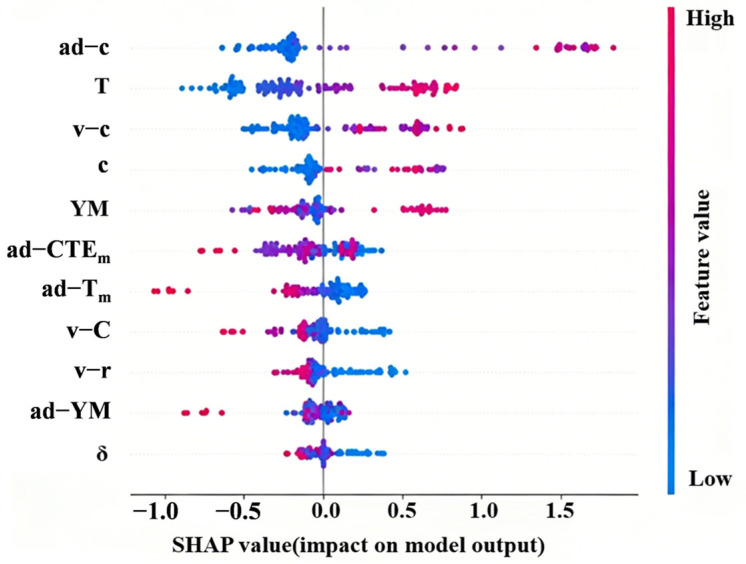
SHAP value distribution of different samples.

Figure 6 presents the SHAP feature importance ranking and distribution based on the RF model. In the figure, the horizontal axis (SHAP value) represents the direction and magnitude of each feature’s influence on the CTE prediction: positive values indicate that the feature promotes CTE, while negative values indicate suppression. The vertical axis is arranged in descending order of global feature importance. The color of each scatter point maps the original value of the corresponding feature (red for high values, blue for low values). From the horizontal distribution width of the features, it can be seen that the c and its derived parameters (*ad*-*c*, *v*-*c*), *T* and *YM* have the most significant impact on the model output. They are the key physical parameters governing the thermal expansion behavior of multi-component alloys.

**Figure 7:**
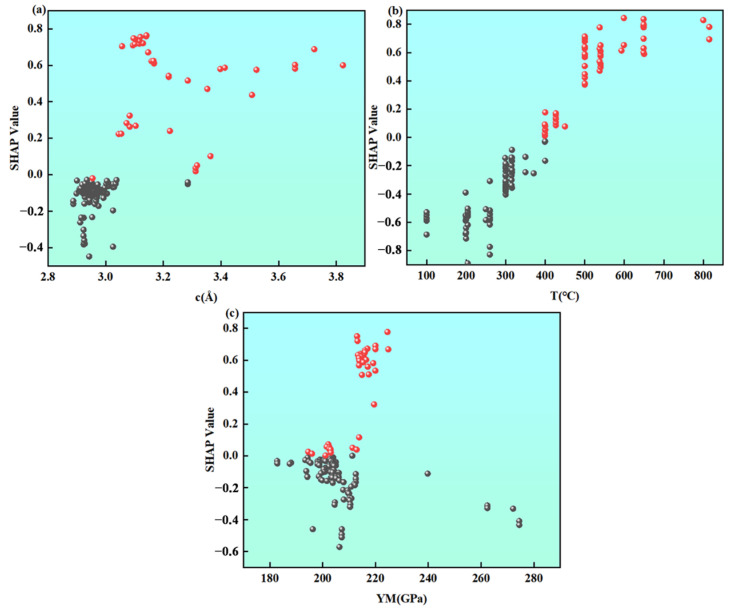
The SHAP value changes with: (**a**) c, (**b**) Tm, (**c**) YM.

Based on the SHAP analysis results, the structure–property relationships of the above core features are discussed in depth one by one. (1) Effect of lattice constant: SHAP analysis reveals a significant positive correlation between the lattice constant *c* and CTE. As shown in Figure 6, when *c* increases, the SHAP value rises accordingly, indicating that its positive contribution to CTE continuously strengthens. This phenomenon can be attributed to two mechanisms. First, a larger c value corresponds to wider atomic spacing, which leads to a reduction in interatomic bonding energy and weakens the lattice’s resistance to thermal expansion. Second, under weak bonding conditions, the anharmonicity of atomic thermal vibrations is enhanced, which intensifies the isotropic volume expansion effect of the lattice. Figure 7a further reveals a critical threshold behavior of *c*. When *c* > 3.04 Å, the SHAP values are predominantly positive, indicating that a larger lattice constant positively drives CTE. When *c* < 3.04 Å, the SHAP values are distributed in the negative range, reflecting that a smaller lattice constant suppresses CTE. Notably, alloy compositions with low *c* values (*c* < 3.04 Å) commonly contain elements such as Ta, V, and Cr. These elements inherently possess small lattice constants. Their low thermal expansion characteristics have been confirmed by experimental studies. (2) Effect of temperature parameter: The temperature parameter exhibits a monotonic positive driving effect on CTE in the model. As shown in Figure 7b, within the temperature range of 110 °C to 800 °C, the SHAP value shows a systematic monotonic increasing trend with rising temperature. It transitions from negative to positive values around 400 °C. This trend indicates that as temperature increases, the positive contribution of the temperature feature to the CTE prediction continuously strengthens. The weak inhibitory effect at low temperatures gradually transforms into a significant promoting effect at high temperatures. This trend is consistent with the classical theory of solid thermal expansion. Increasing temperature leads to a larger mean square displacement of lattice atoms and enhanced anharmonic vibrations, thereby driving volume expansion at the macroscopic scale [44,45,46]. (3) Effect of Young’s modulus: Young’s modulus, as a measure of macroscopic stiffness, exhibits a markedly nonlinear influence on CTE. The SHAP dependence shown in Figure 7c indicates that within the *YM* range of approximately 215–230 GPa, the SHAP values are predominantly positive. This reveals that, in this stiffness range, a higher elastic modulus is unexpectedly accompanied by an increase in CTE. When *YM* > 230 GPa, the SHAP values systematically become negative and continue to decrease as the modulus increases. This transition indicates that after the material stiffness exceeds a critical threshold, the inhibitory effect of strong bonding characteristics on thermally induced expansion gradually becomes dominant. This non-linear stiffness–expansion coupling mechanism reflects that the relationship between elastic constants and the CTE is not a simple monotonic one. Instead, it is a complex physical process governed by the synergistic regulation of lattice vibration anharmonicity and electronic structure.

### 3.4. Sensitivity Analysis of Alloying Elements to the CTE

To establish a quantitative basis for the design of interlayers in dissimilar material joints, this study systematically analyzed the influence of compositional variations of each alloying element on the CTE. The analysis was performed using the trained RF model and took the Fe-C-Si-Cr-Ni-Mn-Mo multi-component alloy system as the object. The results were then used to guide the composition design of the interlayer between 316L stainless steel and Norem02 alloy. A two-step strategy was adopted to determine the composition of the interlayer. First, a preliminary screening was carried out by predicting the CTE of powder mixtures with different proportions. Second, the key compositions were confirmed based on the elemental sensitivity analysis.

#### 3.4.1. Sensitivity Analysis of Each Element to CTE

**Figure 8:**
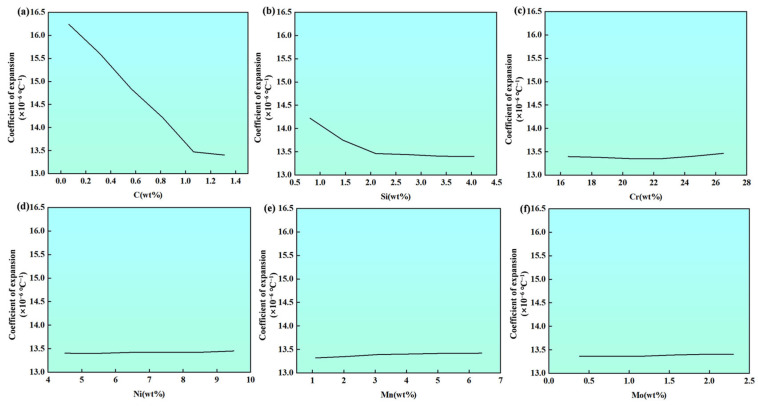
Quantitative analysis of the coefficient of thermal expansion: (**a**) C, (**b**) Si, (**c**) Cr, (**d**) Ni, (**e**) Mn, (**f**) Mo.

The analysis results shown in Figure 8 indicate that the thermal expansion behavior is mainly governed by the contents of C and Si. With increasing C and Si contents, the CTE of the alloy exhibits a clear decreasing trend. This phenomenon is primarily attributed to two factors: the enhancement of interatomic bonding energy in the matrix by C and Si, and their influence on the phase transformation behavior. Together, they effectively suppress the volume expansion of the alloy during heating. In contrast, variations in the contents of elements such as Fe, Cr, Ni, Mn and Mo have a relatively small overall impact on the CTE. Their contributions are rather limited. Therefore, when designing alloys, the contents of C and Si should be precisely controlled as key parameters for tuning the thermal expansion characteristics.

#### 3.4.2. Composition Design of the Interlayer

The CTE values of 316L stainless steel and Norem02 alloy in the range of 20–300 °C are 17.5015 × 10^−6^ °C^−1^ and 13.5251 × 10^−6^ °C^−1^, respectively. To alleviate this mismatch, this study adopted a powder mixing ratio prediction method to preliminarily determine the interlayer composition. Specifically, 316L and Norem02 powders were mixed at different mass ratios (0–100%, where 0% represents pure Norem02 and 100% represents pure 316L). The trained RF model was then used to predict the CTE corresponding to each mixing ratio. The results are shown in Table 3.

As shown in Table 3, the CTE increases monotonically as the Norem02 proportion decreases. When the mixing ratio is 60% 316L + 40% Norem02, the predicted CTE is 15.23 × 10^−6^ °C^−1^. At this ratio, the CTE difference between the interlayer and Norem02 is 1.83 × 10^−6^ °C^−1^, and that between the interlayer and 316L is 2.27 × 10^−6^ °C^−1^. The maximum mismatch is 2.27 × 10^−6^ °C^−1^. Compared with the direct joining case, this mixing ratio reduces the CTE mismatch by about 45%, exhibiting the best CTE mismatch mitigation effect among all mixing ratios examined. Although the 60% mixing ratio already significantly reduces the CTE mismatch, this study further fine-tuned the interlayer composition to further reduce the residual stress. By appropriately adjusting the C content, the thermal expansion matching performance of the interlayer can be further optimized. The detailed composition of the final interlayer is listed in Table 4.

#### 3.4.3. Experimental Validation of the Interlayer Bonding Interface

**Figure 9:**
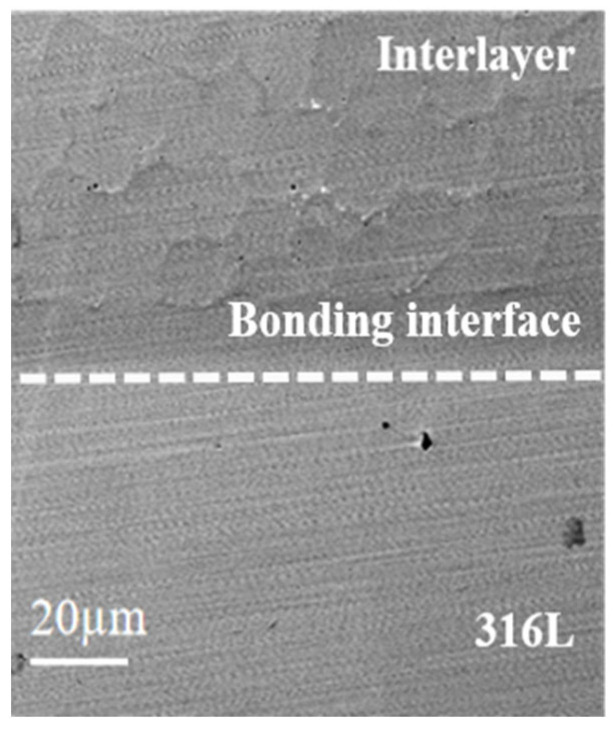
SEM image of the interface between the interlayer and the base material.

To verify the actual joining effect of the designed interlayer, alloy samples were prepared by laser cladding according to the composition listed in Table 4. Figure 9 shows the scanning electron microscopy (SEM) image of the bonding interface between the interlayer and the base metal. The results show that the interlayer is tightly bonded to the base material, and no cracks or delamination defects are observed. This defect-free joining state indicates that the gradient composition designed by the machine learning method can effectively reduce the degree of CTE mismatch, thereby suppressing interfacial failure. The above results validate the engineering feasibility of the machine learning-assisted composition design method from the perspective of joint integrity.

**Figure 10:**
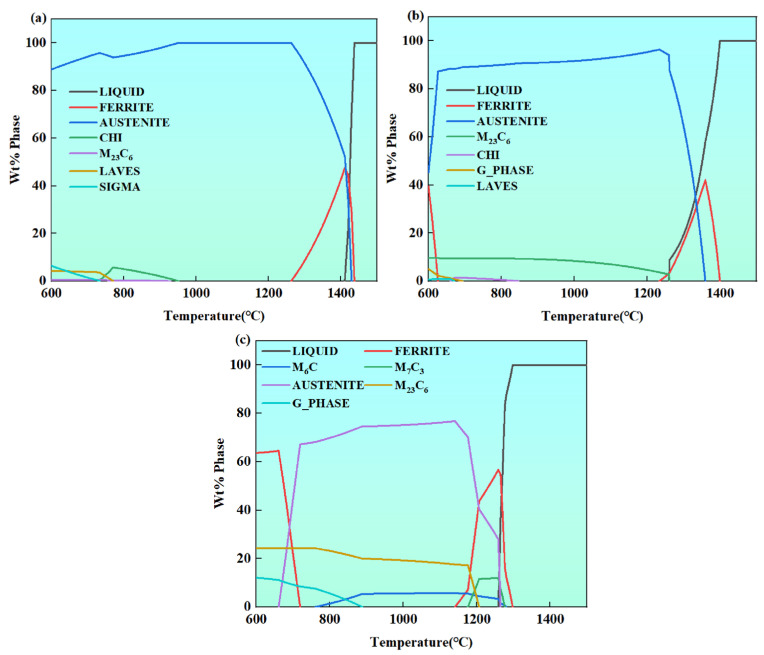
Phase constitutions of 316L, interlayer, and Norem02 calculated by JMatPro: (**a**) 316L, (**b**) Interlayer, (**c**) Norem02.

In functionally graded materials, the phase constitution is a key microstructural factor determining the thermal expansion behavior. Therefore, tailoring the phase constitution is an important approach to alleviating thermal mismatch at dissimilar material interfaces. Among the important features selected by the machine learning model in this study, *VEC*, *δ*, and Δ*H_mix_* are classical criteria for determining phase formation and phase stability of alloys. To further verify the applicability of this study to the present gradient transition layer, the phase constitutions of the 316L substrate, the Norem02 substrate, and the designed interlayer were calculated using the JMatPro thermodynamic software (version 7.0, Sente Software Ltd., Surrey, UK). The calculation results are shown in Figure 10.

As shown in Figure 10, 316L stainless steel exhibits a predominantly austenitic structure. Norem02 is mainly composed of ferrite and M_23_C_6_ carbides. The phase constitution of the interlayer consists of an austenite matrix, ferrite, and a small amount of M_23_C_6_ carbides. Thus, the phase constitution of the interlayer lies between that of 316L and Norem02, forming a clear gradient transition in phase structure. The above phase constitution analysis further demonstrates the effectiveness of the gradient composition designed by the machine learning method, which reduces the degree of interfacial CTE mismatch and suppresses failure behaviors such as cracking and delamination.

To further confirm the effectiveness of the interlayer from the perspective of full-field stress evolution, comparative simulations were performed for two cases: with and without the transition layer. The laser additive manufacturing process was simulated using the Abaqus software (version 2023, Dassault Systèmes Simulia Corp, Johnston, RI, USA), employing a sequentially coupled approach combined with the element birth-and-death technique.

**Figure 11:**
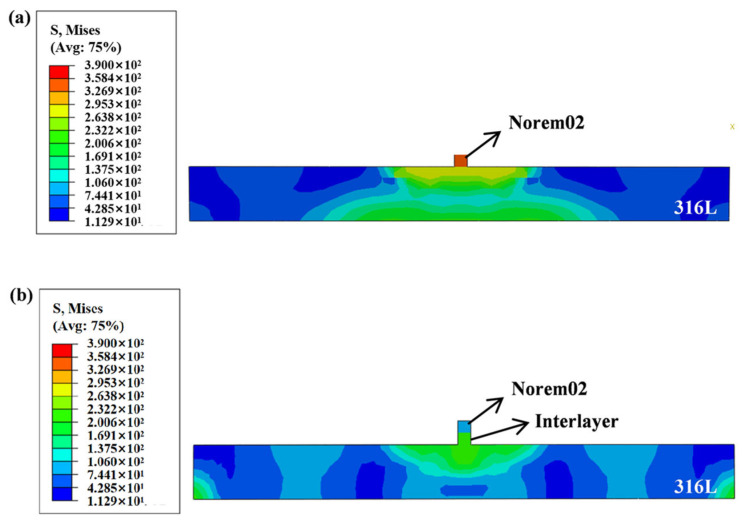
Effect of the addition of a transition layer on residual stress distribution: (**a**) without transition layer, (**b**) with transition layer.

Figure 11 compares the residual stress distribution characteristics with and without the transition layer. Figure 11a corresponds to the case without a transition layer, while Figure 11b shows the simulation result after introducing the transition layer. The stress contour shows that, without a transition layer, significant stress exists in the Norem02 layer. This is attributed to the severe thermal expansion mismatch caused by the large difference in physical property parameters between the substrate and the cladding layer, which leads to the accumulation of high residual stress within the Norem02 layer. After introducing the transition layer, the stress value in the Norem02 layer is significantly reduced. This simulation result indicates that the designed interlayer can effectively buffer the thermal expansion mismatch between the substrate and the functional layer, thereby significantly reducing the residual stress in the Norem02 layer.

**Figure 12:**
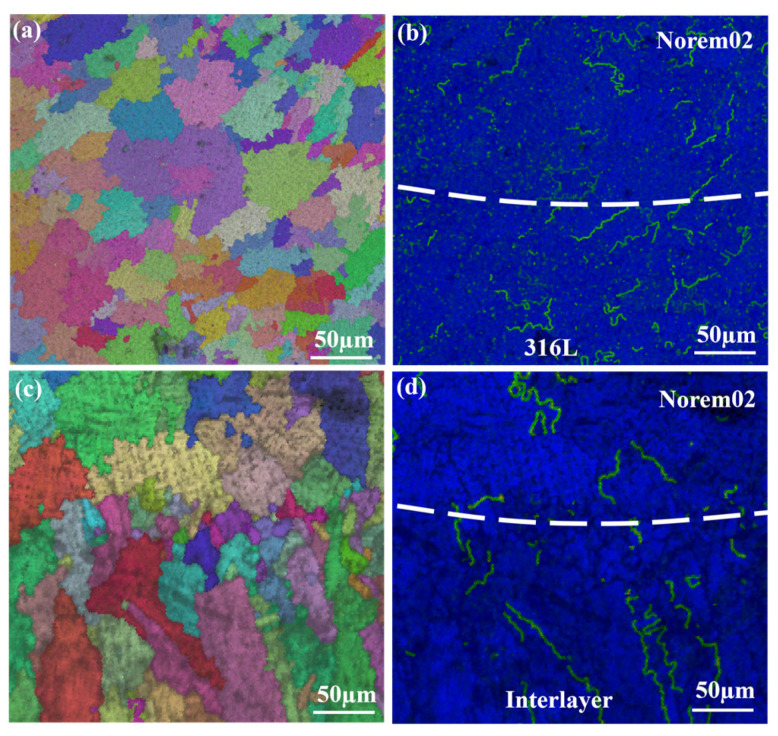
Effect of the addition of a transition layer on residual stress distribution: (**a**) IPF map without transition layer, (**b**) KAM map without transition layer, (**c**) IPF map with transition layer, (**d**) KAM map with transition layer.

The study further employed electron backscatter diffraction (EBSD) to corroborate the above conclusions from an experimental perspective. Figure 12 presents the inverse pole figures (IPF, Figure 12a,c) and the kernel average misorientation (KAM, Figure 12b,d) near the interface, both with and without the transition layer. The KAM maps indirectly characterize the residual strain level in the interfacial region through the distribution of local misorientation. Figure 12b shows the KAM map for the direct joining of 316L and Norem02, while Figure 12d presents the KAM map after introducing the interlayer. A comparison reveals that the KAM value of the Norem02 layer is significantly reduced after the introduction of the interlayer. This indicates that the designed interlayer enables a gradual transition of the coefficient of thermal expansion, thereby reducing the residual strain level in the Norem02 layer.

Together, the above results confirm that the interlayer designed by the machine learning method effectively alleviates the thermal mismatch at the dissimilar material interface, suppresses the accumulation of residual stress, and ensures the integrity of the bonded interface. This strongly validates the feasibility of the machine learning-assisted composition design method in engineering applications.

## 4. Conclusions

This study addresses the CTE mismatch problem at dissimilar material interfaces. A data-driven, machine learning-assisted composition design method is proposed and validated. By systematically constructing a multi-component alloy dataset, optimizing feature engineering, and comparing different models, a high-precision CTE prediction model was established. Combined with interpretability analysis, the influence mechanisms of key physical factors were revealed. The main conclusions are as follows:

(1) A high-precision CTE prediction model based on the RF algorithm was successfully constructed. Using 747 data points covering 15 elements and a three-step feature selection strategy, 11 key feature parameters were screened from 42 initial descriptors. The model achieved an *R*^2^ of 0.929 and an *RMSE* of 0.658 on the test set. Taking Norem02 alloy as the validation object, the average deviation between the predicted and experimental values was only 0.89%. This fully confirms the accuracy and engineering reliability of the model for CTE prediction in multi-component alloy systems.
(2) The interpretability analysis based on the SHAP method indicates that the lattice constant, Young’s modulus, and temperature are the key features affecting the CTE of multi-component alloys. This further reveals the core physical mechanism governing the thermal expansion behavior of these alloys. This mechanism confirms that the feature-property mapping captured by the data-driven model is highly consistent with the intrinsic physical logic of solid thermal expansion, thereby effectively validating the reliability and interpretability of the model. The above findings provide a direct theoretical basis for optimizing the CTE of alloys through compositional regulation.
(3) The feasibility of machine learning-driven composition design for FGMs was validated. The proposed machine learning-assisted design framework overcomes the traditional composition development model that relies on empirical trial-and-error. It enables rapid and accurate prediction of CTE for multi-component alloys and quantitative regulation analysis of key alloying elements (e.g., C and Si). This work not only provides a reliable theoretical basis and technical pathway for the rational design and performance optimization of gradient transition layers at dissimilar material interfaces, but also offers a methodological reference for the data-driven development of other high-performance alloy systems.

## Figures and Tables

**Table 1:** Chemical Composition of 316L Stainless Steel and Norem02 (wt.%).

	C	Cr	Mo	Si	Mn	Ni	Fe
316L	0.025	16.56	2.05	0.606	1.32	10.03	69.409
Norem02	1.31	24.5	2.3	3.4	4.1	4.5	59.89

**Table 2:** Comparison of predicted and experimental average coefficients of thermal expansion of Norem02 alloy in the temperature range of 20–300 °C.

Alloy	Predicted CTE (×10^−6^ °C^−1^)	Experiment CTE (×10^−6^ °C^−1^)	Bias/%
Norem02	13.4034	13.5251	0.89

**Table 3:** Prediction of thermal expansion coefficient of powder mixtures with different mass fractions using machine learning models.

Header	C (wt.%)	Si (wt.%)	Cr (wt.%)	Ni (wt.%)	Mn (wt.%)	Mo (wt.%)	Fe (wt.%)	CTE (×10^−6^ °C^−1^)
0%	1.31	3.4	24.5	4.5	4.1	2.3	59.89	13.4034
10%	1.1815	3.1206	23.706	5.053	3.822	2.275	60.8419	13.4195
20%	1.053	2.8412	22.912	5.606	3.544	2.25	61.7938	13.4378
30%	0.9245	2.5618	22.118	6.159	3.266	2.225	62.7457	13.6861
40%	0.796	2.2824	21.324	6.712	2.988	2.2	63.6976	13.9165
50%	0.6675	2.003	20.53	7.265	2.71	2.175	64.6495	14.6841
60%	0.539	1.7236	19.736	7.818	2.432	2.15	65.6014	15.2313
70%	0.4105	1.4442	18.942	8.371	2.154	2.125	66.5533	15.7683
80%	0.282	1.1648	18.148	8.924	1.876	2.1	67.5052	16.1173
90%	0.1535	0.8854	17.354	9.477	1.598	2.075	68.4571	16.6664
100%	0.025	0.606	16.56	10.03	1.32	2.05	69.409	17.5015

**Table 4:** Composition design and predicted CTE values of the interlayer between 316L and Norem02.

	C (wt.%)	Si (wt.%)	Cr (wt.%)	Ni (wt.%)	Mn (wt.%)	Mo (wt.%)	Fe (wt.%)	CTE (×10^−6^ °C^−1^)
0%	1.31	3.4	24.5	4.5	4.1	2.3	59.89	13.4034
Interlayer	0.5	1.7236	19.736	7.818	2.432	2.15	65.6404	15.3940
100%	0.025	0.606	16.56	10.03	1.32	2.05	69.409	17.5015

## Data Availability

The original contributions presented in this study are included in the article and Supplementary Materials. Further inquiries can be directed to the corresponding author.

## Supplementary Materials

The following supporting information can be downloaded at: https://www.mdpi.com/article/10.3390/ma19102174/s1, Table S1: Common features in alloys; Table S2: The initial feature descriptors and calculation formulas in ML; Table S3: Hyperparameter search ranges for model optimization; Table S4: Optimal feature subset of the model. The references cited in this supplementary file are listed in the main reference list as [24,47,48,49].
