# Supplementary material for: Machine Learning-Based Composition Design of Functionally Graded Alloys
Source: Materials (Basel). 2026 May 21;19(10):2174. doi: 10.3390/ma19102174 (PMC13208268; doi:10.3390/ma19102174)
Supplement: Supplementary file 1 [file materials-19-02174-s001.zip › materials-4291140-supplementary.pdf]

Supplementary information

Part I Basic physical parameters of alloying elements

Table S1. Common features in alloys.

	**C**	**Si**	**Cr**	**Ni**	**Mo**	**Mn**	**Fe**	**Al**	**Co**	**Cu**	**W**	**Nb**	**Ta**	**V**	**Ti**
Tm	3550	1410	1857	1453	2610	1244	1538	660	1495	1083	3422	2468	2966	1890	1688
r	0.77	1.17	1.25	1.24	1.41	1.29	1.26	1.43	1.25	1.28	1.37	1.36	1.43	1.31	1.5
Z	6	14	24	28	42	25	26	13	27	29	74	41	73	23	22
M	12.01	28.09	52	58.69	95.94	54.94	55.85	26.98	58.93	63.55	183.8	92.91	180.9	50.94	47.88
CTE	3.1	2.4	6.2	14.1	5.4	23	11.1	23.2	13	18.2	4.5	7.1	6.3	8.4	8.6
YM	35	155	250	207	329	298	205	70	210	130	344.7	104	186	128	102
ρ	2.26	2.33	7.19	8.9	10.28	7.44	7.87	2.7	8.9	8.96	19.25	8.57	16.69	6.11	4.5
C	720	700	450	460	260	460	449	897	385	386	130	260	140	480	520
a	3.57	5.43	2.8665	3.524	3.16	8.91	2.866	4.049	2.507	3.614	3.165	3.300	3.301	3.026	2.9508
b	3.57	5.43	2.8665	3.524	3.16	8.91	2.866	4.049	2.507	3.614	3.165	3.300	3.301	3.026	2.9508
c	3.57	5.43	2.8665	4.524	3.16	8.91	2.866	4.049	4.199	3.614	3.165	3.300	3.031	3.026	4.6824
VEC	4	4	6	10	6	7	8	3	9	11	6	5	5	5	4
χ	2.55	1.9	1.66	1.91	2.16	1.55	1.83	1.61	1.88	1.9	2.36	1.6	1.5	1.63	1.54

Table S1 is a commonly used characteristic of alloys. Among them, T_m is the melting point (K), *r* is the atomic radius (Å), *Z* is the atomic number, *M* is the relative atomic mass, *CTE* is the linear thermal expansion coefficient of the alloy (10^{-6} ℃$^{-1}$), *YM* is the Young's modulus (GPa), *C* is the specific heat capacity (J/(kg·K)), *a*, *b*, *c* are the lattice constants of atoms (Å), *VEC* is the valence electron concentration, and χ is the electronegativity.

Part Ⅱ Feature Descriptor Construction

In the field of machine learning, feature selection and construction undoubtedly occupy a pivotal position. These features are not randomly generated, but carefully selected from physical parameters that are closely related to the prediction target. These physical parameters carry rich information and can directly or indirectly reflect the intrinsic characteristics of the prediction target. To maximize the performance of machine learning models, we often choose the weighted average, absolute deviation, and variance of physical parameters as feature descriptors[47]. The weighted average helps us capture the average performance of parameters over a period of time or under certain conditions. The absolute deviation reveals the deviation degree between parameter values and the average, thus reflecting the stability and consistency of the data. Variance further provides a measure of the fluctuation of parameter values, enabling us to gain a more comprehensive understanding of the data distribution. Here, we combine the main physical parameters in Table S1 and calculate the weighted

average, absolute deviation, and variance to obtain 42 descriptors in Table S2.

Table S2. The initial feature descriptors and calculation formulas in ML.

No.	Phase parameter	Formula	Description
1	ΔH_{mix}	Previous work within the group (improved Miedema model and Chou model)	mixing enthalpy**Error! Reference source not found.**
2	ΔS_{mix}	Previous work within the group (improved Miedema model and Chou model)	configuration entropy**Error! Reference source not found.**
3	ΔG_{mix}	Previous work within the group (improved Miedema model and Chou model)	Gibbs Free Energy Change**Error! Reference source not found.**
4	δ	$\sqrt{\sum_{i=1}^{n} C_i \left(1-\frac{r_i}{r}\right)^2}$	atomic size difference **Error! Reference source not found.**
5	χ	$\sum_{i=1}^{n} C_i \chi_i$	pauling electronegativity **Error! Reference source not found.**
6	T_m	$\sum_{i=1}^{n} C_i T_{mi}$	melting point **Error! Reference source not found.**
7	$ad\text{-}T_m$	$\sum_{i=1}^{n} C_i \left\|T_{mi} - T_m\right\|$	absolute deviation of melting point
8	$v\text{-}T_m$	$\frac{\sum_{i=1}^{n} C_i \left\|T_{mi} - T_m\right\|}{n-1}$	variance of melting point
9	r	$\sum_{i=1}^{n} C_i r_i$	atom radius **Error! Reference source not found.**
10	$ad\text{-}r$	$\sum_{i=1}^{n} C_i \| r_i - r \|^2$	absolute deviation of atom radius
11	$v\text{-}r$	$\frac{\sum_{i=1}^{n} C_i \| r_i - r \|^2}{n-1}$	variance of atom radius
12	Z	$\sum_{i=1}^{n} C_i Z_i$	atomic number
13	$ad\text{-}Z$	$\sum_{i=1}^{n} C_i \| Z_i - Z \|$	absolute deviation of atomic number
14	$v\text{-}Z$	$\frac{\sum_{i=1}^{n} C_i \| Z_i - Z \|^2}{n-1}$	variance of atomic number
15	M	$\sum_{i=1}^{n} C_i M_i$	relative atomic mass

16	*ad-M*	$\sum_{i=1}^{n} C_i \mid M_i - M \mid$	absolute deviation of atomic number
17	*v-M*	$\frac{\sum_{i=1}^{n} C_i \mid M_i - M \mid^2}{n-1}$	variance of relative atomic mass
18	*CTEm*	$\sum_{i=1}^{n} C_i CTE_{mi}$	Thermal expansion coefficient
19	*ad-CTEm*	$\sum_{i=1}^{n} C_i \mid CTE_{mi} - CTE_m \mid$	absolute deviation of Thermal expansion coefficient
20	*v-CTEm*	$\frac{\sum_{i=1}^{n} C_i \mid CTE_{mi} - CTE_m \mid^2}{n-1}$	variance of Thermal expansion coefficient
21	*YM*	$\sum_{i=1}^{n} C_i YM_i$	Young's modulus
22	*ad-YM*	$\sum_{i=1}^{n} C_i \mid YM_i - YM \mid$	absolute deviation of Young's modulus
23	*v-YM*	$\frac{\sum_{i=1}^{n} C_i \mid YM_i - YM \mid^2}{n-1}$	variance of Young's modulus
24	*ρ*	$\sum_{i=1}^{n} C_i \rho_i$	density
25	*ad-ρ*	$\sum_{i=1}^{n} C_i \mid \rho_i - \rho \mid$	absolute deviation of density
26	*v-ρ*	$\frac{\sum_{i=1}^{n} C_i \mid \rho_{\mathrm{i}} - \rho \mid^2}{n-1}$	variance of density
27	C	$\sum_{i=1}^{n} C_i C$	Specific heat capacity
28	*ad-C*	$\sum_{i=1}^{n} C_i \mid C_{\mathrm{i}} - C \mid^2$	absolute deviation of Specific heat capacity
29	*v-C*	$\frac{\sum_{i=1}^{n} C_i \mid C_{\mathrm{i}} - C \mid^2}{n-1}$	variance of Specific heat capacity
30	*a*	$\sum_{i=1}^{n} C_i a_i$	Lattice constant a
31	*ad-a*	$\sum_{i=1}^{n} C_i \mid a_{\mathrm{i}} - a \mid$	absolute deviation of Lattice constant a
32	*v-a*	$\frac{\sum_{i=1}^{n} C_i \mid a_{\mathrm{i}} - a \mid^2}{n-1}$	variance of Lattice constant a
33	*b*	$\sum_{i=1}^{n} C_i b_i$	Lattice constant b

34	*ad-b*	$\sum_{i=1}^{n} C_i \mid b_{\mathrm{i}} - b \mid$	absolute deviation of Lattice constant b
35	*v-b*	$\frac{\sum_{i=1}^{n} C_i \mid b_{\mathrm{i}} - b \mid^2}{n-1}$	variance of Lattice constant b
36	*c*	$\sum_{i=1}^{n} C_i c_i$	Lattice constant c
37	*ad-c*	$\sum_{i=1}^{n} C_i \mid c_i - c \mid$	absolute deviation of Lattice constant c
38	*v-c*	$\frac{\sum_{i=1}^{n} C_i \mid C_i - c \mid^2}{n-1}$	variance of Lattice constant c
39	*VEC*	$\sum_{i=1}^{n} C_i VEC_i$	valence electron concentration**Error! Reference source not found.**
40	*ad-VEC*	$\sum_{i=1}^{n} C_i \mid VEC_i - VEC \mid$	absolute deviation of valence electron concentration
41	*v-VEC*	$\frac{\sum_{i=1}^{n} C_i \mid VEC_i - VEC \mid^2}{n-1}$	variance of valence electron concentration
42	*Ω*	$T_{\mathrm{m}} * \frac{\Delta S_i}{\mid \Delta H_i \mid}$	entropic ratio parameter *Ω*

Notes: The letter with the subscript i indicates the physical properties of an element in the alloys.

Part Ⅲ Machine learning model hyperparameter setting

To systematically evaluate the performance of different machine learning algorithms, we performed hyperparameter optimization on six candidate models. A 10-fold cross-validation approach was adopted to identify the optimal parameter combinations. The search space for each algorithm is defined as follows:

Table S3. Hyperparameter search ranges for model optimization.

Algorithms	Hyperparameter search ranges for model optimization
RF	param_grid = {'n_estimators': [int(x) for x in np.linspace(start = 100, stop =3000, num= 3)],<br>'max_features': ['auto', 'sqrt'],<br>'max_depth': [int(x) for x in np.linspace(10, 110, num =3)],<br>'min_samples_split': [2,5],<br>'min_samples_leaf': [1,2],<br>'bootstrap': [True]}
LR	ridge_tuned_parameters = {<br>'ridge__alpha': [0.1, 1.0, 10.0, 20,30,50,60,80,100.0],<br>'ridge__solver': ['liblinear', 'saga', 'newton-cg', 'lbfgs', 'sag'],<br>'ridge__tol': [1e-4, 1e-3, 1e-2] }
MLP	param_grid = {

	'hidden_layer_sizes': [(100,), (50, 50), (100, 100)],<br>'activation': ['tanh', 'relu'],<br>'solver': ['sgd', 'adam'],<br>'alpha': [0.0001, 0.001,0.01,0.1],<br>'batch_size': ['auto', 128, 256],<br>'learning_rate': ['constant', 'adaptive'],<br>'learning_rate_init': [0.001, 0.01],<br>'max_iter': [200, 500,1000]<br>}
KNN	knn_tuned_parameters = {<br>'n_neighbors': range(1, 100),<br>'weights': ['uniform', 'distance'],<br>'p': [1, 2]<br>}
NN	nn_clf__tuned_parameters = {<br>'hidden_layer_sizes': [(5,5,5,5,5),(50, 50),(30, 30),(60,30,15),(20, 20), (10, 10), (20,20,10),],<br>'activation': ['relu', 'tanh'],<br>'alpha': [0.0001, 0.001, 0.01],<br>'learning_rate': ['constant', 'adaptive'],<br>}
ReNet	kfold = KFold(n_splits=10, shuffle=True, random_state=1)<br>learning_rates = [0.001,0.05,0.08,0.1,]<br>batch_sizes = [32,16,8,24,64]

Part Ⅳ Optimal feature subset of the model.

Following a three-step feature selection procedure, the optimal feature subsets identified for each machine learning algorithm are summarized in Table S4.

Table S4. Optimal feature subset of the model.

Algorithms	Optimal feature subset
KNN	$YM,c,v\text{-}c,ad\text{-}CTE_{m},ad\text{-}c,T$
RF	$ad\text{-}T_{m},\delta,YM,c,v\text{-}r,v\text{-}C,v\text{-}c,ad\text{-}CTE_{m},ad\text{-}YM,ad\text{-}c,T$
LR	$\delta,YM,c,v\text{-}r,v\text{-}C,v\text{-}c,ad\text{-}CTE_{m},ad\text{-}YM,ad\text{-}c,T$
NN	$ad\text{-}T_{m},\delta,YM,c,v\text{-}r,v\text{-}C,v\text{-}c,ad\text{-}CTE_{m},ad\text{-}YM,ad\text{-}c,T$
MLP	$\delta,YM,c,v\text{-}r,v\text{-}C,v\text{-}c,v\text{-}VEC,ad\text{-}T_{m},ad\text{-}CTE_{m},ad\text{-}YM,ad\text{-}c,T$
ReNet	$YM,c,v\text{-}r,v\text{-}C,v\text{-}c,ad\text{-}CTE_{m},ad\text{-}c,T$

References

All references cited in this supplementary file are listed in the main reference list of the article (references [24,47–49]).
